# Regular weighing to prevent excessive gestational weight gain: a study protocol for a systematic review and meta-analysis

**DOI:** 10.1186/s13643-022-01977-6

**Published:** 2022-05-26

**Authors:** Tomomi Matsushita, Aiko Honda, Takeshi Hasegawa, Eisuke Inoue, Hisashi Noma, Erika Ota

**Affiliations:** 1 Department of Obstetrics and Gynecology, Showa University School of Medicine, 1-5-8 Hatanodai, Shinagawa-Ku, Tokyo, 142-8555 Japan; 2 Department of Pediatrics, Showa University Hospital, 1-5-8 Hatanodai, Shinagawa-Ku, Tokyo, 142-8555 Japan; 3 Showa University Research Administration Center (SURAC); Department of Hygiene, Public Health, and Preventive Medicine, School of Medicine; Division of Nephrology, Department of Medicine, School of Medicine, Showa University, 1-5-8 Hatanodai, Shinagawa-Ku, Tokyo, 142-8555 Japan; 4 Showa University Research Administration Center, Showa University, 1-5-8 Hatanodai, Shinagawa-Ku, Tokyo, 142-8555 Japan; 5 Department of Data Science, The Institute of Statistical Mathematics, 10-3 Midori-cho, Tachikawa, Tokyo, 190-8562 Japan; 6 St. Luke’s International University, Graduate School of Nursing, Global Health Nursing, 10-1 Akashi-cho, Chuo-ku, Tokyo, 104-0044 Japan; 7 Tokyo Foundation for Policy Research, Tokyo, Japan

**Keywords:** Gestational weight gain, Antenatal care, Weighing in pregnancy, Gestational diabetes, Macrosomia

## Abstract

**Background:**

Excessive weight gain during pregnancy results in maternal and fetal complications and could further impact offspring. The evidence regarding the association between regular weighing during the antenatal period and excessive weight gain is limited.

**Methods:**

We will systematically review individual and cluster randomized controlled trials that evaluated regular weighing as an intervention compared to weighing only at the first booking of the antenatal visit. Trials that assessed the effectiveness of exercise, diet, or other behavioral interventions will be excluded. Pregnant women with a singleton pregnancy and no preexisting health complications are eligible for the review. The primary outcome will be the proportion of women at term who exceed the upper limit of the target range of weight as defined by the guidelines or recommendations for the population. We will search MEDLINE (via PubMed), Embase (via EMBASE.com), Scopus, the Cumulative Index to Nursing and Allied Health Literature (CINAHL via EBSCO), The Cochrane Central Register of Controlled Trials (CENTRAL) and the trial protocol registers, ClinicalTrials.gov, and the International Clinical Trials Registry Platform (ICTRP) search portal. Full-text articles, unpublished studies, and ongoing trials reported in any language will be included. Two review authors will independently examine and screen for eligible studies and extract data for synthesis.

**Discussion:**

We will discuss the effectiveness of regular weighing as a single intervention on reducing the proportion of women who have excessive gestational weight gain. This study will provide key information for countries to develop guidelines on antenatal care and strategies to tackle excessive gestational weight gain. We will create a “Summary of findings” table (Summary of findings table 1) according to the methods described in the Cochrane Handbook for Systematic Reviews of Interventions.

**Systematic review registration:**

PROSPERO CRD42020212581

**Supplementary Information:**

The online version contains supplementary material available at 10.1186/s13643-022-01977-6.

## Background

### Description of the condition

#### Gestational weight gain and its health outcomes

Optimal weight gain during pregnancy has long been investigated. The guidelines from the Institute of Medicine (IOM) published in 2009 [1] are widely adopted in many national guidelines, including Australia and New Zealand [2], the UK [3], and Canada [4]. The IOM guidelines set recommended weight gains for pregnant women with four different weight statuses according to prepregnancy body mass index (BMI), namely, underweight (BMI less than 18.5), normal weight (BMI between 18.5 and 24.9), overweight (BMI between 25.0 and 29.9), and obese (BMI 30.0 or more) [1]. The range of recommended weight gain for women in the underweight group is 12.5 to 18kg. As prepregnancy BMI increases, the range of recommended weight gain narrows. For women in the normal weight, overweight, and obese groups, the weight-gain ranges are 11.5 to 16kg, 6.8 to 11.3kg, and 5 to 9.1kg, respectively. Anyone who gains more than the upper limit of the recommended weight range is defined as having gestational excessive gestational weight gain (GWG), and anyone who does not achieve the lower limit of the recommended range is defined as having inadequate weight gain in pregnancy [1].

GWG is related to various maternal and neonatal health outcomes. Excessive GWG in particular is a public health concern that increases the rate of large for gestational age, macrosomia, preeclampsia, gestational diabetes, and cesarean delivery [5–7]. In the USA, excessive GWG affects close to 50% of pregnant women [8, 9], with overweight and obese women having the highest prevalence [8]. The percentages are similar across other countries: 38% in Australia [10], 53.8% in France [11], and 47% worldwide according to a recent systematic review that examined 23 studies [12]. Several factors are associated with the increased risk of excessive GWG, including being overweight or obese according to prepregnancy BMI, smoking cessation during pregnancy [13], and women’s misperception of prepregnancy BMI [14]. Moreover, research revealed epigenetic impact on offspring of mothers who developed high-glucose tolerance status during pregnancy (namely high BMI, gestational diabetes, and excessive GWG), including increased incidence of childhood overweight and obesity [15, 16]. Excessive weight gain in pregnancy also impacts long-term BMI after birth [17, 18].

### Description of the intervention

#### Prevention of excessive gestational weight gain

Behavioral health interventions have been the focus of excessive GWG prevention. Interventions that promote exercise, healthy eating, or both have shown effectiveness in reducing the incidence of excessive GWG [19–21]. Nevertheless, lifestyle interventions rely on behavioral changes that some pregnant women may find it challenging to maintain [22].

#### Antenatal visits and weighing

Antenatal visits aim to provide an essential guide for improving perinatal outcomes for mothers and babies. Pregnant women visit medical personnel 4 to 12 times for check-ups, depending on their country’s policies. Antenatal visits start as early as 4 weeks of pregnancy, when women recognize that they have conceived. At the first visits, many countries’ guidelines recommend checking women’s body parameters, including height, weight, and BMI calculated by the former two parameters [2–4]. In some places, weighing is a routine practice on every antenatal visit. In others, after the first antenatal visit, women are weighed only if clinical management requires their current weight [3, 23].

#### Regular or repeated weighing at antenatal visit

In a country where weighing pregnant women is routine care, pregnant women receive guidance to check their weight regularly, usually at every antenatal visit. To measure weight as accurately as possible, health providers have women wear light clothing and no shoes when weighing. Health providers track the weight and advise women if necessary, according to the target weight gain based on prepregnancy BMI groups [4].

#### Regular or repeated weighing at home

People weigh themselves at home with a digital or analog scale to monitor their weight regularly for a set period. They record their weight in a diary or an application on their phones. On a clinic visit or through the application, they receive feedback from health care providers on their weight progress in relation to the maximum and minimum limits [24].

### How the intervention might work

Weighing, as a way of self-monitoring, has been effective in optimizing weights among the non-pregnant population. In recent randomized controlled trials, self-weighing, daily or weekly, depending on the trials, showed the effect on reducing the proportion of people who gained or reduced weight, compared to control arms without self-weighing [25, 26]. The mechanism of its effectiveness lies in self-regulatory and monitoring theory, in which individuals become aware of weight changes and also can observe and assess the impact of their lifestyle patterns on the changes, ultimately promoting motivation for behavioral change [24, 27].

### Why is it important to do this review?

As obesity and overweight become an epidemic, close to one third of pregnant women fall into one of these categories when they become pregnant [4, 28]. As prepregnancy BMI is a single important risk factor for excessive GWG, effective interventions to prevent excessive GWG in pregnancy are needed. Moreover, regardless of prepregnancy BMI, appropriate weight gain is critical for better perinatal outcomes [5]. In a recent systematic review, exercise or diet interventions, or both, showed a 20% reduction of the risk of excessive GWG [19]. Both exercise and diet interventions hold the underlying assumption of behavioral change by patients, and that is the limitation of these interventions. In other words, patients have to change their behavioral patterns for the intervention to be effective. To date, evidence of interventions other than physical activity and food intake is lacking. In 2017, one systematic review examined the impact of weighing pregnant women as a stand-alone intervention, and no difference was found between the intervention and control groups [29]. However, the review only included trials published in English, possibly limiting the number of trials included in the analysis. Moreover, there may be new studies published since the last review. In this review, we will aim to update the evidence regarding the weighing of pregnant women regularly through antenatal periods to reduce excessive GWG.

## Methods/design

### Protocol development

We followed the PRISMA statement [30] as we developed the present protocol. We first registered this protocol in PROSPERO with the registration number CRD42020212581. All the recommended reporting items in the PRISMA statement were addressed in this protocol (see Additional file 1 for PRISMA-P+checklist). We will conduct this systematic review in accordance with the Cochrane Handbook for Systematic Reviews of Interventions [31].

### Criteria for considering studies for this review

The detailed criteria for inclusion and exclusion of studies are described in Additional file 2.

#### Types of studies

We will include individual and cluster randomized controlled trials comparing regular weighing to only weighing at the initial booking of antenatal check-ups. To help reduce bias, we will exclude quasi-randomized or other experimental trials.

#### Types of participants

The participants will be pregnant women with a singleton pregnancy and no preexisting health complications.

#### Types of interventions

##### Intervention

Any regular or repeated weight measurement either by a woman herself or by a health care provider. Weighing can be done either at home or at a health care facility. Interventions that combine regular weighing with other lifestyle interventions, including diet or exercise, will be excluded. If a study provided an intervention that included a behavioral intervention that may affect GWG, other than weighing, the study will be excluded. A study will be included when both intervention and control groups received behavioral intervention as part of usual care, and only the intervention group received regular weighing.

##### Control

Weight measurement at the first booking of the antenatal visit, at the trial enrollment, or both. Women will receive no further instructions for weighing, either at home or at a health care facility. Weighing can be repeated only when clinically required.

### Types of outcome measures

#### Primary outcome

The proportion of women who had excessive GWG (e.g., the proportion of women who exceed the upper limit of the target weight range as defined by the guidelines or recommendations for the population).

#### Secondary outcome

- Incidence of large for gestational age (>90th percentile of birthweight for sex and gestational age)
- Incidence of macrosomia (birthweight >4000g)
- Rate of Apgar Score <7 at 5 min
- Rate of cesarean delivery
- Incidence of preeclampsia
- Incidence of gestational diabetes
- Incidence of maternal and postpartum depression

We will create a “Summary of findings” table (Summary of findings table 1) on the following seven outcomes.

1. The proportion of women at term who exceeds the upper limit of the target range of weight as defined by the guidelines or recommendations for the population.
2. Incidence of large for gestational age (>90th percentile of birthweight for sex and gestational age)
3. Incidence of macrosomia (birthweight >4000g)
4. Rate of Apgar Score <7 at 5 min
5. Rate of cesarean delivery
6. Incidence of preeclampsia
7. Incidence of maternal and postpartum depression

### Search methods for identification of studies

We will electronically search MEDLINE (via PubMed), Embase (via EMBASE.com), Scopus, the Cumulative Index to Nursing and Allied Health Literature (CINAHL via EBSCO), The Cochrane Central Register of Controlled Trials (CENTRAL) and the trial protocol registers, ClinicalTrials.gov, and the International Clinical Trials Registry Platform (ICTRP) search portal for published, unpublished, and ongoing studies with no restrictions on language or publication date. We will minimize missing data by contacting authors where possible. If the obtained data is incomplete, we will address it as one of our study limitations. Information specialists will create the search strategies for each database based on the draft search strategy for PubMed (see Additional file 3).

### Data collection

#### Selection of studies

We will identify records through the database using prespecified search terms and then pool the results in Endnote as citations. After removing duplicates, two review authors (TM, AH) will independently examine the remaining references. We will exclude studies that do not meet the inclusion criteria (File 1). Full-text records of potentially relevant references will then be obtained and imported into Endnote. Review authors (TM, AH) will independently screen the eligibility of the retrieved records, extract data in a predetermined form, and exclude ineligible records. Any disagreement will be resolved by the third author (TH). A list of studies that we excluded after a full-text review will be provided in the excluded studies table, with the reason for exclusion. We will create a diagram (the PRISMA flowchart) for the study selection process.

#### Data extraction and management

For eligible studies, we will extract the following information in a predetermined form.

- Author, published year, title, and the journal
- Trial periods
- Country
- Study settings (e.g., urban or rural area, hospital, or clinic)
- Study design
- Definition of excessive weight gain
- Follow-up period
- Participants
  - Number of participants
  - Eligible criteria, exclusion criteria
  - Method of baseline weight measurement
  - Total number of participants in each study arm
- Interventions
  - Frequency of weighing
  - Place of weighing
  - Weighing protocol
  - Weighing device
  - Method of weight recording
  - Intervention other than weighing
- Control details
- Outcomes
  - Primary outcome
  - Secondary outcome
  - Definition of outcomes
  - Unit of measurement
  - Time points assessed
- Analytical methods
- Results
- Others
  - Funding status
    Two review authors (TM, AH) will independently extract data on the form and any disagreement will be discussed and referred to the third author (TH) if necessary.

### Assessment of risk of bias in included studies

We will use the following the Cochrane Collaboration’s tool criteria to assess the risk of bias in included studies. Two review authors (TM, AH) will independently assess the criteria, and any disagreement will be resolved by involving the third author (TH).

1. Random sequence generation
2. Allocation concealment
3. Blinding of participants and personnel
4. Outcome assessment
5. Incomplete outcome data
6. Selective reporting
7. Other bias (checking for bias due to problems not covered by [1] to [5] above)

We will explore the effect of the study bias on the finding through sensitivity analysis (see Sensitivity analysis).

### Strategy for data synthesis

Summary data from each randomized controlled trial will be pooled into a meta-analysis when adequate, comparable data are available. We will use the Review Manager (RevMan) software, version 5.4 (Cochrane Collaboration, Oxford, UK), for the synthesis analyses. If we cannot perform quantitative synthesis, we will analyze and describe the results.

#### Measures of treatment effect

For measures of treatment effect, we will use the risk difference or risk ratio, as appropriate.

#### Dealing with missing data

We will assess the impact of missing data through sensitivity analysis if a significant proportion of data is missing.

#### Assessment of heterogeneity

For the synthesis analyses, we will apply the random-effects model to address the between-studies heterogeneity. Statistical heterogeneity among the trials will be evaluated by the heterogeneity variance *τ*^2^, Higgins’ *I*^2^ statistic, and *Q* statistic (Cochrane’s test). The significance level of the Cochrane’s test will be set at 0.10. To assess between-studies heterogeneity by potential effect modifiers, we will conduct subgroup analyses or meta-regression analyses.

#### Assessment of reporting biases

We will create funnel plots and visually assess potential reporting biases. Also, we will conduct publication bias tests to evaluate small-study effects.

#### Subgroup analysis

To assess potential sources of heterogeneity, we will conduct subgroup analyses for the following factors: paras (nulliparous versus multiparous), prepregnancy BMI categories, weighing frequency (e.g., daily versus less frequent than daily), and self-weighing versus weighing by health care providers. If the definitions of excessive weight gain in the included studies are different, we will consider a subgroup analysis by stratifying included studies into groups according to the definitions of excessive weight gain.

#### Sensitivity analysis

We will conduct sensitivity analyses to assess the influence of trial quality by removing those trials assessed as “high risk” or “unclear” bias regarding random sequence generation (selection bias) and incomplete outcome data (attrition bias). We will use only our primary outcome for sensitivity analysis.

#### Summary of findings and assessment of the certainty of the evidence

We will present the overall certainty of the evidence for the following seven outcomes based on the Grading of Recommendations Assessment, Development and Evaluation (GRADE) approach. We will create a “Summary of findings” table (Summary of findings table 1) according to the methods described in the Cochrane Handbook for Systematic Reviews of Interventions:

1. The proportion of women at term who exceeds the upper limit of the target range of weight as defined by the guidelines or recommendations for the population.
2. Incidence of large for gestational age (>90th percentile of birthweight for sex and gestational age)
3. Incidence of macrosomia (birthweight >4000g)
4. Rate of Apgar Score <7 at 5 min
5. Rate of cesarean delivery
6. Incidence of preeclampsia
7. Incidence of maternal and postpartum depression

## Discussion

This systematic review protocol reports methodological steps in the review process to examine the current data regarding the effectiveness of regular weighing as part of antenatal care on excessive GWG. The review has several limitations. First, regular weighing is routine in some countries, but rarer in others [23]. The contextual difference of routine care may impact the intervention’s effect. Second, this study is susceptible to between-studies heterogeneity expected for the difference in settings with a different physical profile of women and national guidance on GWG. Although BMI categories applied in IOM guidelines are widely adapted, previous research suggested that applying regional BMI categories for GWG is more appropriate for assessing pregnancy outcomes [5]. That potential heterogeneity in included studies can make it difficult to produce an overall summary in a meta-analysis of pooled estimates for excessive weight gain on maternal and infant outcomes. Third, biases through the process of estimating weight gain during pregnancy are expected. Getting a baseline weight may lead to potential measurement biases. If a self-reported prepregnancy weight is used as a surrogate measure of baseline weight, the baseline weight might be underestimated. If the weight at the first antenatal booking is used as a baseline weight, that might not reflect a true prepregnancy weight. Last, although we will exclude studies other than randomized controlled trials to minimize bias, blinding of participants and personnel is expected to be ranked as “high risk” or “unclear” bias because of the nature of the intervention. The quality of our findings will potentially be limited due to the performance bias.

This study and its meta-analysis aim to explore a strategy to tackle excessive GWG that is affordable, efficient, and adaptable to existing services. After synthesizing the findings of this systematic review, we will report the results in a peer-reviewed journal and present them at scientific meetings. The findings will provide insights for setting guidelines on weight measurement during the antenatal period.

## Supplementary information

**Additional file 1.** PRISMA-P+ checklist.

**Additional file 2.** Eligibility criteria.

**Additional file 3.** Search strategy.

## Data Availability

Not applicable

## **Abbreviations**

IOM: The Institute of Medicine
BMI: Body mass index
GWG: Gestational weight gain
CENTRAL: The Cochrane Central Register of Controlled Trials
CINAHL: Cumulative Index to Nursing and Allied Health Literature
ICTRP: The International Clinical Trials Registry Platform
GRADE: The Grading of Recommendations Assessment, Development and Evaluation
PROSPERO: International Prospective Register of Systematic Reviews
PRISMA-P: Preferred Reporting Items for Systematic Review and Meta-analysis Protocols
PRISMA: Preferred Reporting Items for Systematic Review and Meta-analysis
