# Supplementary material for: Regular weighing to prevent excessive gestational weight gain: a study protocol for a systematic review and meta-analysis
Source: Syst Rev. 2022 May 26;11:104. doi: 10.1186/s13643-022-01977-6 (PMC9137211; doi:10.1186/s13643-022-01977-6)
Supplement: Supplementary file 2 — Additional file 2. Eligibility criteria. [file 13643_2022_1977_MOESM2_ESM.docx]

Additional File 2: Specific study characteristics for eligibility for the review

Inclusion and Exclusion Criteria

|  | Inclusion Criteria | Exclusion Criteria |
| --- | --- | --- |
| Participants | - Pregnant women with no age restriction - Singleton pregnancy | - Pregnant women with preexisting health complications that require regular weight monitoring, including diabetes, hypertension, or renal disease |
| Intervention | - Regular or repeated weight measurement exclusive of other diet or exercise interventions - Weighing done either by a woman herself or by a health care provider | - Interventions that combine regular weighing with other lifestyle interventions - Interventions that include a behavioural intervention that may affect gestational weight gain, other than weighing |
| Comparison/  Control Group | - No instructions for regular or repeated weighing - Only weighing at the first booking of the antenatal visit or the study enrollment | - Repeated weighing without a clinical requirement |
| Outcome of Interest | - Trials reporting at least one of the outcomes | - Weight loss |
| Study Design | - Individual and cluster randomised controlled trials - Full-text articles published in a peer-reviewed journal - Conference abstracts or other grey literature - No language or year restrictions | - Study designs other than randomised controlled trials |
| Setting/Context | - No country restrictions - Antenatal visits in any settings |  |
