# Supplementary material for: Regular weighing to prevent excessive gestational weight gain: a study protocol for a systematic review and meta-analysis
Source: Syst Rev. 2022 May 26;11:104. doi: 10.1186/s13643-022-01977-6 (PMC9137211; doi:10.1186/s13643-022-01977-6)
Supplement: Supplementary file 3 — Additional file 3. Search strategy. [file 13643_2022_1977_MOESM3_ESM.docx]

Additional File 3: Search Strategy

PubMed search

| #1 | ("Pregnancy"[Mesh:NoExp] OR "Gravidity"[Mesh] OR "pregnant women"[MeSH Terms] OR "Gestational Weight Gain"[Mesh] OR pregnant[tiab] OR pregnanc*[tiab] OR expectant-mother*[tiab] OR during-gestation[tiab] OR gestational-period*[tiab] OR gestational-weight*[tiab] OR gestational-body*[tiab] OR gravidit*[tiab]) |
| --- | --- |
| #2 | "body weight changes"[MeSH:noexp] OR "body weight maintenance"[MeSH Terms] OR "weighing"[tiab] OR weight-monitor*[tiab] OR weight-gain-monit*[tiab] OR weight-control*[tiab] OR weight-maint*[tiab] OR weight-manag*[tiab] OR weight-measur*[tiab] OR weight-chang*[tiab] OR ((bodyweight*[tiab] OR weight*[tiab]) AND (regular-measur*[tiab] OR daily-measur*[tiab] OR routine-measur*[tiab])) |
| #3 | (("Controlled Clinical Trial"[Publication Type] OR "randomized"[Title/Abstract] OR "randomised"[Title/Abstract] OR "Clinical Trials as Topic"[MeSH Terms:noexp] OR "placebo"[Title/Abstract] OR "randomly"[Title/Abstract] OR "trial"[Title]) NOT ("animals"[MeSH Terms] NOT "humans"[MeSH Terms])) |
| #4 | #1 AND #2 AND #3 |

CENTRAL (via Wiley) search

| #1 | (pregnanc* OR pregnant OR gravidit* OR ((during OR period* OR weight* OR body*) NEAR/3 gestation*) OR (expectant-mother*)):ti,ab,kw |
| --- | --- |
| #2 | (weighing OR ((weight* OR bodyweight* OR BW) NEAR/3 (maint* OR manag* OR measur* OR chang* OR monitor* OR daily OR weekly OR regular* OR routin*)) OR ((weight* OR bodyweight* OR BW) NEAR/2 control*)):ti,ab,kw |
| #3 | #1 AND #2 |
